# Mutations in *HPCA* Cause Autosomal-Recessive Primary Isolated Dystonia

**DOI:** 10.1016/j.ajhg.2015.02.007

**Published:** 2015-03-19

**Authors:** Gavin Charlesworth, Plamena R. Angelova, Fernando Bartolomé-Robledo, Mina Ryten, Daniah Trabzuni, Maria Stamelou, Andrey Y. Abramov, Kailash P. Bhatia, Nicholas W. Wood

**Affiliations:** 1 Department of Molecular Neuroscience, UCL Institute of Neurology, Queen Square, London WC1N 3BG, UK; 2 Department of Medical and Molecular Genetics, King’s College London, London WC2R 2LS, UK; 3 Department of Genetics, King Faisal Specialist Hospital and Research Centre, PO Box 3354, Riyadh 11211, Saudi Arabia; 4 Sobell Department of Motor Neuroscience and Movement Disorders, UCL Institute of Neurology, Queen Square, London WC1N 3BG, UK; 5 Second Department of Neurology, University of Athens, Iras 39, Gerakas Attikis, Athens 15344, Greece; 6 Movement Disorders Department, Hygeia Hospital, 4 Eyrthrou Stravou Street, Athens 15123, Greece; 7 UCL Genetics Institute, London WC1E 6BT, UK

## Abstract

Reports of primary isolated dystonia inherited in an autosomal-recessive (AR) manner, often lumped together as “DYT2 dystonia,” have appeared in the scientific literature for several decades, but no genetic cause has been identified to date. Using a combination of homozygosity mapping and whole-exome sequencing in a consanguineous kindred affected by AR isolated dystonia, we identified homozygous mutations in *HPCA*, a gene encoding a neuronal calcium sensor protein found almost exclusively in the brain and at particularly high levels in the striatum, as the cause of disease in this family. Subsequently, compound-heterozygous mutations in *HPCA* were also identified in a second independent kindred affected by AR isolated dystonia. Functional studies suggest that hippocalcin might play a role in regulating voltage-dependent calcium channels. The identification of mutations in *HPCA* as a cause of AR primary isolated dystonia paves the way for further studies to assess whether “DYT2 dystonia” is a genetically homogeneous condition or not.

## Main Text

Dystonia is a common movement disorder characterized by twisting or repetitive movements with or without tremor.^1^ Dystonia occurring with no other neurological signs on clinical examination and normal neuroimaging is currently classified as “primary isolated dystonia.” Mendelian forms of this primary isolated dystonia have been associated with several genes (*TOR1A* [MIM 605204],^2^ *THAP1* [MIM 609250],^3^ *CIZ1* [MIM 611420],^4^ *ANO3* [MIM 610110],^5^ *GNAL* [MIM 139312],^6^ and *TUBB4A* [MIM 602662]^7^, ^8^), all of which are inherited in an autosomal-dominant fashion. Previous reports have nonetheless suggested that an autosomal-recessive (AR) form of primary isolated dystonia might exist. In general, these reports have been lumped together under the banner of DYT2 (MIM 224500) or “DYT2-like” dystonia.^9^, ^10^, ^11^, ^12^ DYT2 is thus somewhat of an anomaly given that it is defined purely by phenotype and presumed mode of inheritance without any associated linkage interval. The sole exception to this practice was the creation in 2008 of the DYT17 locus to designate a chromosome 20 region, defined by homozygosity mapping, in a consanguineous Lebanese kindred exhibiting isolated AR dystonia.^13^ Yet, as with DYT2, the genetic cause remains to be identified.^13^

**Figure 1:**
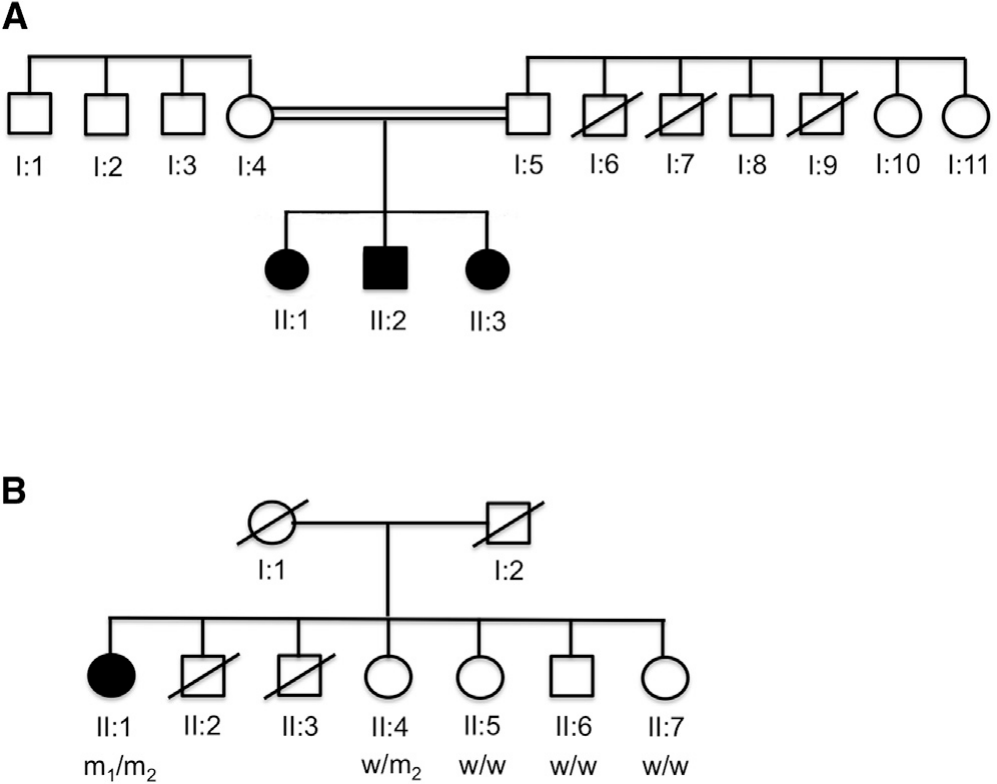
Genetic Pedigrees for Families Affected by Mutations in *HPCA* Abbreviated genetic pedigrees are shown for the core members of (A) the index family and (B) the second family identified to be affected by compound-heterozygous mutations in *HPCA*. For the family in (B), the results of the segregation analysis are shown under each individual: WT, wild-type allele; M_1_, c.212C>A (p.Thr71Asn) mutation; M_2_, c.568G>C (Ala190Thr) mutation. The results are consistent with AR inheritance of dystonia due to biallelic mutations in *HPCA*. Individual II:6 did not report any symptoms suggestive of dystonia; however, this could not be confirmed by examination because he did not live in the UK.

With the advent of next-generation sequencing technologies, we revisited a Sephardic Jewish kindred exhibiting AR isolated dystonia that had previously been classified as “DYT2-like.”^10^ The three affected siblings (now aged 61, 57, and 51 years) were the product of a consanguineous marriage between two first cousins. Both parents were neurologically normal, and there was no report of any dystonia within the wider kindred (Figure 1A and extended pedigree in Khan et al.^10^). In brief, in their first decade of life, all three affected siblings developed dystonia, which gradually generalized over time but remained most marked in the upper limbs and cervical and cranial regions. Initially, the siblings were reported to have an atypical form of metachromatic leukodystrophy (MLD) on the basis of markedly reduced levels of arylsulfatase A in fibroblasts and leucocytes, reduced nerve conduction velocities, and the detection of brown metachromatic granules in sural nerve biopsies. Subsequent mutational screening by Sanger sequencing demonstrated that the mother and the three siblings were homozygous and that the father was heterozygous for two variants in a *cis* configuration, c.[1055A>G; ^∗^96A>G] (RefSeq accession number NM_000487.5), which are commonly referred to collectively as the “polyA mutation,” in the gene *ARSA* (MIM 607574). The polyA mutation results in reduced amounts of arylsulfatase A on biochemical assay and has no clinical symptoms (a state termed pseudodeficiency). No other mutations were detected in the remainder of the gene at that time or in the current study. In this context, the detection of metachromatic granules in the sural nerve biopsy is unusual. Nonetheless, despite prolonged follow-up, no clinical or radiological features of progressive central or peripheral demyelination have developed, making MLD highly unlikely. On current examination, there are no other neurological features besides the dystonia detectable on clinical examination, and exhaustive radiological and biochemical investigations have failed to reveal any underlying cause. Extensive genetic testing—including but not confined to *TOR1A*, *THAP1*, *GNAL*, and *ANO3*—has not revealed a causal mutation in these genes. Although the severity of the dystonia has gradually increased over time, the clinical course appears relatively benign: all three of the affected siblings continue to function well in daily life, there is no significant limitation of ambulation, and fixed deformities have not developed. This study was approved by the relevant local ethics committee at our institution, and informed consent was provided by all participants in accordance with its guidelines.

DNA was extracted from whole-blood samples obtained from all three affected siblings and both parents. DNA from one affected sibling was used for performing whole-exome sequencing with Illumina’s TruSeq (62 Mb) DNA sample preparation and exome enrichment kits. With the TruSeq exome definition as a reference, coverage was 96% at a read depth of 2×, 87% at a read depth of 10×, and 74% at a read depth of 20×. The mean read depth across the exome was 58×. In total, 22,097 variants were detected.

Genome-wide genotyping data were generated with the OmniExpress platform (∼500,000 markers) and used for performing homozygosity mapping. Tracts of potential homozygosity greater than 1 Mb were initially identified for each individual. The boundaries of overlapping homozygosity tracts shared by all three affected individuals were subsequently determined. Coverage across regions of shared homozygosity was calculated with BedTools against the Consensus Coding Sequence (CCDS) definition of the exome and was expressed as the percentage of target bases covered by at least one read (see Table 1). Mean coverage of CCDS genes across all homozygous regions was 90.7%.

In view of the apparently recessive inheritance pattern and history of consanguinity, we initially selected all homozygous variants for consideration. Subsequently, synonymous variants not predicted to affect splicing (i.e., those not within ten bases, in either direction, of the intron-exon boundary) were discarded. Given the rarity of AR isolated dystonia, we further hypothesized that the causal variant would not be found in any database of normal sequence variation. However, in order to minimize the possibility of incorrectly assigning causality, we filtered out only those variants at a minor allele frequency of greater than 0.5%. Given that a variant found at even this frequency would be expected to occur naturally in the homozygous state in around 1 in every 160,000 births, it seemed distinctly unlikely that we would risk filtering out the causal variant with this cutoff. Finally, variants that were located in regions of shared homozygosity were selected as potentially causal (see Table 1). No filtration was performed on the basis of in silico predictions of pathogenicity or conservation scores.

Filtering of the exome data as above left just two possible candidate causal homozygous variants (both located in the largest stretch of shared homozygosity on chromosome 1) for further consideration: the first (c.625G>A [p.Val209Met]; RefSeq NM_006762.2) was in exon 7 of *LAPTM5* (MIM 601476), which encodes a lysosomal transmembrane protein, and the second (c.225C>A [p.Asn75Lys]; RefSeq NM_002143.2) was in exon 2 of *HPCA* (MIM 142622), which encodes the neuronal calcium sensor (NCS) protein known as hippocalcin.

**Figure 2:**
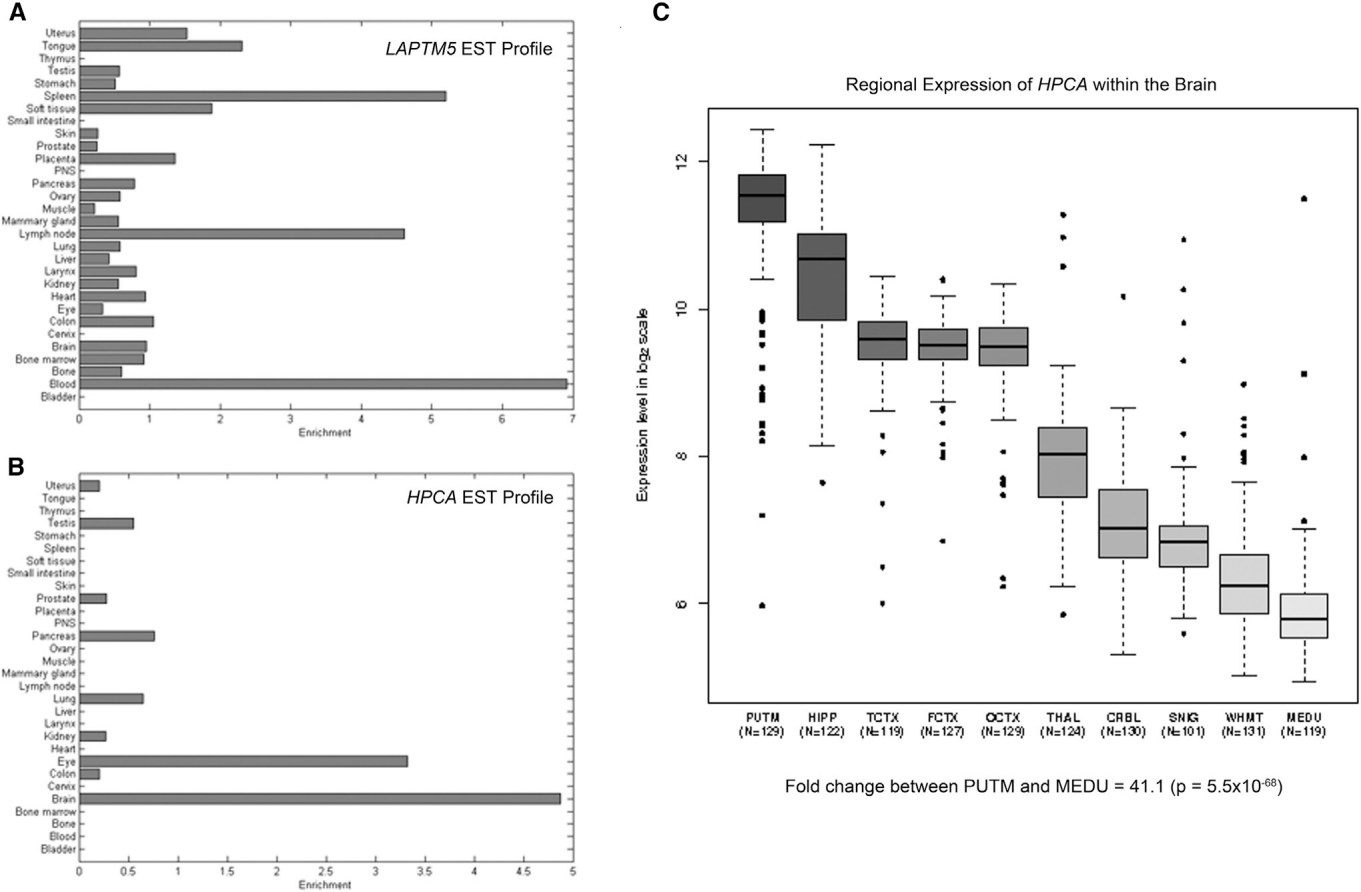
Expression Data for *LAPTM5* and *HPCA* (A and B) Publically available expressed sequence tag (EST) data for (A) *LAPTM5* and (B) *HPCA* demonstrate that both genes show relatively tissue-specific expression patterns. *LAPTM5* is predominantly expressed in hemopoietic tissues, whereas *HPCA* is almost exclusively expressed in the brain. (C) Boxplot of mRNA expression levels for *HPCA* in ten CNS regions. Data are based on in-house exon array experiments and plotted on a log_2_ scale (y axis). A full description of the samples used, the methods of RNA isolation and processing, and data-analysis steps can be found in Trabzuni et al.^14^ This plot shows significant variation in *HPCA* transcript expression across the ten CNS regions analyzed: putamen (PUTM, n = 129), hippocampus (HIPP, n = 122), temporal cortex (TCTX, n = 119), frontal cortex (FCTX, n = 127), occipital cortex (OCTX, n = 129), thalamus (THAL, n = 124), cerebellar cortex (CRBL, n = 130), substantia nigra (SNIG, n = 101), intralobular white matter (WHMT, n = 131), and medulla (specifically inferior olivary nucleus, MEDU, n = 109). *HPCA* mRNA expression is highest in the putamen, followed closely by the hippocampus. Expression is also high in the cortex. Whiskers extend from the box to 1.5× the inter-quartile range.

To aid in determining which was the most plausible candidate, we obtained (1) in silico predictions of pathogenicity from SIFT, PROVEAN, PolyPhen-2, and MutationTaster and (2) expression data for each gene, and we assessed the location of the detected variants in relation to predicted protein functional domains (see Table 2 and Figure 2). Although the variants in both genes showed high levels of conservation and were universally predicted to be damaging, the variant in *HPCA* appeared to be the stronger candidate because (1) the variant affected a key functional domain of the protein and (2) the gene is almost exclusively expressed in the brain—particularly in the striatum, an area connected to movement disorders—whereas *LAPTM5* is expressed poorly in the brain and most avidly in tissues involved in hemopoiesis.

In fact, the p.Asn75Lys variant in hippocalcin affects an amino acid that is known to be critical to a key functional domain’s calcium-binding properties. All NCS proteins, including hippocalcin, are characterized by four EF-hand domains that act as potential Ca^2+^ binding sites. However, EF-hand domain 1 is invariably inactive in binding divalent cations, and EF-hand domain 4 is only active in some NCS proteins. Canonical Ca^2+^-binding EF-hand domains are characterized by the semi-conserved sequence motif D-X-D/N-X-D/N-G-(X)_5_-E, where the underlined obligate amino acids are involved in the coordinative binding of Ca^2+^.^15^, ^16^ The p.Asn75Lys variant in hippocalcin results in a substitution of the second Ca^2+^-coordinating residue of the binding sequence within EF-hand domain 2. Usually, only one of two amino acids will be found at this position: a negatively charged aspartic acid or a neutral asparagine (as in wild-type hippocalcin). Unsurprisingly, the amino acid at this position shows absolute interspecies conservation (Figure S1A). Moreover, the p.Asn75Lys variant leads to the incorporation of a positively charged lysine, which might reasonably be expected to cause a particular impediment to the binding of the similarly charged Ca^2+^ ion.

In wild-type NCS proteins, binding of Ca^2+^ to their functional EF-hand domains operates a myristoyl switch mechanism—most extensively studied in the related neural protein recoverin—that controls the protein’s ability to translocate to target membranes and/or interact with downstream effectors (Figure S2).^17^, ^18^, ^19^ By means of this mechanism, NCS proteins, like hippocalcin, are able to act as reversible transducers of cellular Ca^2+^ signals and are thus capable of integrating both temporal and spatial aspects over a tight dynamic range.^17^ Thus, it is plausible to hypothesize that the homozygous p.Asn75Lys variant, by impairing or even preventing Ca^2+^ binding to EF-hand domain 2, would be expected to reduce the likelihood of conformational change of hippocalcin in response to Ca^2+^ signals and, by extension, result in a defect in cellular Ca^2+^ signal transduction in the striatum, an area of the brain intimately connected with movement disorders, such as dystonia.

Despite the greater inherent biological plausibility of the variant in *HPCA*, we next attempted to find further confirmatory mutations in either candidate gene. To do so, we obtained dystonia samples (donated with research consent) from a DNA bank held at our institution. Despite the extensive nature of this clinical resource, the rarity of AR isolated dystonia meant that there were no other samples available from any other dystonia kindred in whom the inheritance pattern could definitively be said to be AR. We were therefore forced instead to select subjects for whom the history was merely “not incompatible” with AR inheritance (i.e., subjects with either no family history or a family history of affected siblings only). All subjects had been screened previously for mutations in *TOR1A*, and no mutations had been identified.

In order to reduce consumption of DNA and protect this valuable clinical resource for future use, we adopted a two-phased screening strategy. In the first phase, we aimed to identify the most likely candidate genes by sequencing only the exons in which the potentially causal variants were identified in the index family in an independent cohort of 150 subjects with young-onset (<30 years of age), non-autosomal-dominant dystonia of any distribution (younger age of onset was prioritized). In addition, we included a DNA sample from an affected member of the DYT17 kindred. We did not detect any further potentially causal variants in exon 7 of *LAPTM5*. In exon 2 of *HPCA*, however, we detected a second, heterozygous, missense variant (c.212C>A) resulting in an amino acid substitution (p.Thr71Asn) at a position just 4 amino acids before the location of the original homozygous variant found in the index family. The affected nucleotide shows extremely high conservation scores (PhyloP = 5.76 [max = 6]; PhastCons = 1 [max = 1]), and the affected amino acid is conserved in all species. Although this amino acid is not recognized as an obligatory Ca^2+^ coordinator itself, it is still within the second EF-hand domain of hippocalcin (amino acids 60–95 according to UniProt) and is predicted to be damaging, with near maximal probability scores, by all four in silico prediction programs.

On the basis of this finding, in the second phase, we went on to sequence all coding exons of *HPCA* in the same 151 samples and also in a second cohort of 288 non-autosomal-dominant, young-onset (<40 years of age) subjects exhibiting dystonia that was either generalized or most prominent in the upper limbs or cervical or cranial region (i.e., we prioritized a distribution similar to the phenotype observed in other families). We found only one additional variant of any kind, and this was in the same sample that harbored the p.Thr71Asn substitution. The additional variant was a missense mutation (c.568G>C [p.Ala190Thr]) located toward the end of *HPCA* exon 4, which encodes the C terminus of the protein. The nucleotide involved is conserved (PhyloP = 2.015; PhastCons = 1), and the affected amino acid is conserved in most species, except the fly (*D. melanogaster*) and the Tasmanian devil (*S. harrisii*). However, only MutationTaster predicts it to be disease causing; SIFT, PROVEAN, and PolyPhen-2 predict that the substitution will be tolerated. The p.Ala190Thr substitution is not located in any EF-hand domain, and the mechanism by which it might impair protein function is less obvious. However, we note that it has previously been suggested that the C-terminal regions of other NCS proteins might be involved in fine-tuning their response or determining target specificity.^20^, ^21^

We established contact with the individual whose DNA sample harbored the compound-heterozygous mutations in *HPCA*—a 64 year-old woman of Sri Lankan origin—to verify the medical and family history and perform a full neurological examination. She reported that the onset of dystonia was in her early twenties, possibly even her late teens. It initially manifested with abnormal involuntary finger movements that were most noticeable when she tried to type. Over time, her dystonia very gradually worsened: a tremulous component emerged, but the dystonia remained segmental, such that it affected only the hands, arms, and muscles of the neck, and would be classified clinically as mild. Despite the fact that she is one of seven siblings, no other family member, including her parents, siblings, and her siblings’ children, reported or were reported to have any symptoms consistent with dystonia, and this was confirmed by clinical examination where possible (Figure 1B). Segregation analysis in the four surviving siblings demonstrated that the remaining unaffected siblings possessed either one or both wild-type alleles (Figure 1B), supporting the pathogenicity of the compound-heterozygous mutations in the affected individual.

It is notable that, with the exception of the single sample detailed above, we did not detect any other coding or splice-site variant—previously annotated or not— in *HPCA* in any of the other subjects screened. This low level of variation is confirmed by the pooled next-generation sequencing results of the NHLBI Exome Sequencing Project, ClinSeq, 1000 Genomes, and the International HapMap Project, which have collectively identified only eight separate missense variants (none of which are homozygous) in the 8,451 individuals of various ethnicities for whom data have so far been made public (Table S1). By way of comparison, on the basis of the same datasets, 31 missense (11 of which are predicted to be damaging by both SIFT and PolyPhen-2), two splice-site, and one frameshift variant have been detected in *LAPTM5*. Although we cannot, because of a lack of data, be certain that this low level of variation in *HPCA* extends to all populations, this observation does at least further increase the likelihood that the identification of compound-heterozygous *HPCA* mutations segregating perfectly with disease in a second dystonia-affected kindred was very unlikely to have occurred by chance.

Given the position and nature of the p.Asn75Lys and p.Thr71Asn variants, we hypothesized that they would lead to a loss of function. We therefore performed short hairpin RNA (shRNA) knockdown of *Hpca* on rat primary neurons and astrocytes and used the resultant cells to measure, by means of fura-2 florescence microscopy, the effect of hippocalcin deficiency on cellular calcium homeostasis after exposure to different neuropharmacological agents.

For primary cortical cultures, *Wistar* WT rat pups were culled between postnatal days 1 and 3, and a primary co-culture was prepared as described elsewhere.^22^ Cerebral hemispheres were trypsinized and resuspended in 2 ml of warm complete Neurobasal A medium, and the cell suspension was plated on poly-L-lysine-coated coverslips. The cultures were incubated at 37°C in a humidified incubator with 5% CO_2_ in the air for 3–4 hr, and then 2 ml of pre-warmed complete Neurobasal A medium was added.

*Hpca* knockdown in rat cortical primary cultures was performed by Effectene transfection (QIAGEN) after 9–10 days in culture with either a pool of four rat-specific shRNAs or individual shRNAs targeting rat *Hpca* (Thermo Fischer). The empty vector (pGIPZ) and the vector expressing a non-targeting RNA (SCR) were used as controls. The transfection was made according to the manufacturer’s instructions, and 48 hr after transfection, the cells were ready for subsequent experiments.

We stimulated the resultant cells with (1) 5 μM glutamate to simulate a physiological calcium signal in neurons via activation of glutamate receptors, (2) 100 μM ATP to stimulate P2Y receptors in astrocytes, and (3) 50 mM potassium chloride (KCl) to depolarize neuronal membranes and induce the opening of voltage-gated calcium channels.

**Figure 3:**
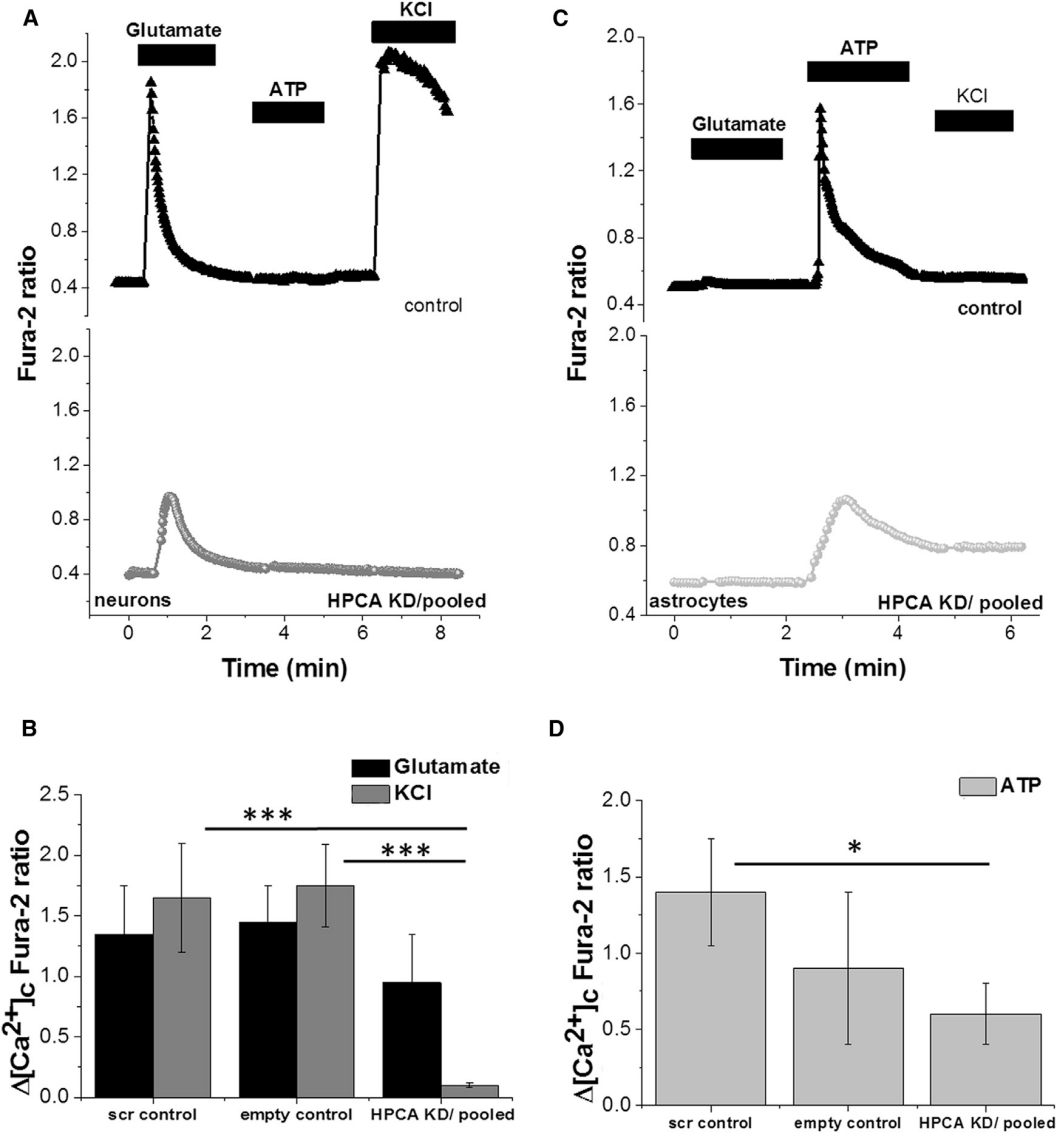
Summary of the Functional Studies in *Hpca*-Knockdown Neuronal-Astrocytic Co-cultures Astrocytes and neurons from primary cortical co-culture were loaded for 30 min at room temperature with 5 μM fura-2 AM and 0.005% pluronic acid in a HEPES-buffered salt solution composed of 156 mM NaCl, 3 mM KCl, 2 mM MgSO_4_, 1.25 mM KH_2_PO_4_, 2 mM CaCl_2_, 10 mM glucose, and 10 mM HEPES (pH was adjusted to 7.35 with NaOH). Fluorescence measurements were obtained on an epifluorescence inverted microscope equipped with a 20× fluorite objective. [Ca^2+^]_c_ was monitored in single cells with excitation light provided by a Xenon arc lamp, and the beam passed through a monochromator at 340 and 380 nm (Cairn Research). Emitted fluorescence light was reflected through a 515-nm longpass filter to a charge-coupled-device camera (Retiga, QImaging) and digitized to a 12-bit resolution. All imaging data were collected and analyzed with software from Andor IQ. The fura-2 data were not calibrated in terms of [Ca^2+^]_c_ because of the uncertainty arising from the use of different calibration techniques. Areas for the analysis were chosen depending on the GFP fluorescence intensity, and four independent experiments were performed for each condition. The figure shows representative traces of [Ca^2+^]_c_ response to physiological stimuli as measured by changes in fura-2 fluorescence intensity. Compared to neurons from the scrambled or empty control (A, black triangle trace), *Hpca*-knockdown neurons showed no rise in [Ca^2+^]_c_ in response to depolarization of the plasma membrane with 50 mM KCl (A, dark-gray trace; B, dark-gray bars). In addition, the amplitude of the response to physiological concentration of glutamate (5 μM) was lower in the *Hpca*-knockdown neurons than in control cells (B, black bars), although this decrease was not statistically significant. *Hpca* knockdown also diminished the amplitude of the [Ca^2+^]_c_ response of astrocytes to an ATP stimulus (100 μM) (C and D, light-gray trace and bars). Error bars represent the SEM, and asterisks represents statistical significance (^∗∗∗^p < 0.0001, ^∗^p < 0.05).

We found that application of glutamate resulted in a smaller, but non-significant, neuronal response to glutamate in *Hpca*-shRNA-transfected neurons (0.95 ± 0.4, n = 35; see Figure 3A, lower panel) than in the controls transfected with scrambled vector (1.35 ± 0.4, n = 35, p = 0.48) and empty vector (1.45 ± 0.3, n = 21, p = 0.38; see Figure 3B, upper panel).

Astrocytic signal to stimulation with 100 μM ATP in *Hpca*-knockdown cells was also smaller (0.6 ± 0.2, n = 55; Figures 3C and 3D) than that in control cells transfected with scrambled vector (1.4 ± 0.35, n = 45) and empty vector (0.9 ± 0.5, n = 28), but the reduction in signal only reached significance when *Hpca*-knockdown cells were compared to scrambled controls (p = 0.04).

Somewhat unexpectedly, the most significant difference between *Hpca*-silenced and control neurons was observed after depolarization of the plasma membrane by application of 50 mM KCL. As anticipated, this resulted in a strong Ca^2+^ signal in control neurons transfected with scrambled vector (1.65 ± 0.45, n = 35) and empty vector (1.75 ± 0.34, n = 21). In *Hpca*-shRNA-transfected neurons, however, application of KCL produced almost no observable Ca^2+^ signal (0.1 ± 0.02, n = 35; p = 0.001 versus controls with scrambled vector, and p < 0.0001 versus controls with empty vector; Figures 3A and 3B). Importantly, all of these cells demonstrated a clear response to glutamate, confirming their neuronal origin (Figure 3B).

Together, this pattern of severely altered neuronal responses to physiological stimuli suggests that *HPCA* deficiency might inhibit voltage-dependent Ca^2+^ channels or, alternatively, modify the mechanism of maintaining the membrane potential and thus affect cellular response to membrane depolarization.

In summary, we have presented evidence to support biallelic mutations in *HPCA* as a cause of AR primary isolated dystonia. This discovery resolves the mystery surrounding the nature of DYT2 dystonia and decisively settles the debates over the existence of an AR form of isolated dystonia. Although we were only able to identify one additional family affected by dystonia secondary to mutations in *HPCA*, this is consistent with the relative rarity of the disorder, and it is unlikely that any European or American institution would have had a significantly higher number of cases. However, screening of further suitable cases, particularly in geographical regions with higher rates of consanguinity, will be needed to give a better idea of its actual prevalence. This should be relatively easy given the small size of the gene.

This identification of variants in hippocalcin, an NCS protein detected in greatest abundance in the striatum, as the cause of a Mendelian form of dystonia suggests a role for perturbed calcium signaling in the pathogenesis of this condition. In terms of future research, it is important to note that NCS proteins, such as hippocalcin, possess no inherent enzymatic properties but exert their Ca^2+^-dependent functions through interactions with other proteins. Thus, the identification of downstream interactors is a necessary first step in understanding their specific activities. In the case of hippocalcin, the identity of its interactors remains incomplete. There is, nonetheless, evidence to suggest that it might have a role in (1) the modulation of cyclic nucleotide signaling in the olfactory epithelium,^23^ (2) long-term depression in the hippocampus,^24^, ^25^, ^26^ (3) generation of the slow afterhyperpolarization current (important in controlling neuronal excitability),^27^, ^28^, ^29^ (4) regulation of gene transcription,^30^, ^31^ and (5) neurite outgrowth.^32^ Although some other, currently unknown function of hippocalcin might underlie its involvement in dystonia, it is notable that, in relation to the processes mentioned above, both aberrant excitability of striatal neurons and altered synaptic plasticity due in part to decreased long-term depression are two mechanisms believed to be important in at least some forms of dystonia.^33^, ^34^, ^35^, ^36^

Hippocalcin has been most intensely studied in relation to its role in synaptic plasticity within the hippocampus, where it is hypothesized to play a role in memory formation. Indeed, *Hpca*-knockout mice show deficits in tests of spatial and associative memory in the absence of any obvious structural abnormalities within the brain.^30^ Difficulties with memory were not, however, reported by any of the individuals in whom we detected *HPCA* mutations, and this study did not have ethical approval or funding to perform detailed neuropsychological testing. However, recent neuropsychological testing performed with consent as part of the medical investigation of one member of the index family did show evidence of cognitive underfunctioning, including particular difficulties in encoding verbal and visual information. Whether memory deficits represent a subtle and variable associated phenotype in humans with *HPCA* mutations (as does hyposmia in those with *GNAL* mutations^37^) or whether the neuropsychological deficits observed in the individual in this study were an incidental finding remains an open question that only the identification of further affected individuals and a study dedicated to their neuropsychological profiling will answer.

## Figures and Tables

**Table 1:** Regions of Homozygosity and Exome Sequencing

**Chr**	**Start**	**End**	**Length (Mb)**	**CCDS Genes**	**Coverage**	**Variants Detected**	**Potentially Causal Variants**
1	12,880,356	20,476,391	7.60	86	84.3%	166	0
1	26,909,765	34,686,130	7.78	102	91.0%	43	2
3	126,380,804	127,502,549	1.12	7	97.9%	5	0
5	98,552,184	99,968,045	1.42	1	100%	0	0
6	34,502,022	36,226,525	1.72	28	95.9%	16	0
7	64,926,823	66,464,764	1.54	10	82.7%	3	0
8	48,639,976	49,656,604	1.02	5	80.5%	3	0
8	85,802,488	86,990,451	1.19	8	89.7%	4	0
11	47,976,882	51,591,253	3.61	13	95.9%	32	0
11	54,794,237	55,943,322	1.15	25	89.4%	52	0

For each homozygous region shared between all three siblings, the table shows genomic coordinates (UCSC Genome Browser hg19), the length (Mb), the number of CCDS genes that lie in the region, the percentage of CCDS bases (including UTRs) covered by exome sequencing, the number of variants detected in that region, and the number of potentially causal variants that remained after filtration, as detailed in the methods. The following abbreviation is used: Chr, chromosome.

**Table 2:** Candidate Causal Variants after Filtration

	**Homozygous Change**	
**c.625G>A (p.Val209Met)**	**c.225C>A (p.Asn75Lys)**
Chromosome	1	1
Position (hg19)	31,208,094	33,354,724
Gene (RefSeq transcript)	*LAPTM5* (NM_006762.2)	*HPCA* (NM_002143.2)
Previously reported	no	no
PhyloP	C (3.37)	C (2.71)
PhastCons	C (1)	C (1)
SIFT	D (0.001)	D (0.001)
PROVEAN	D (−2.7)	D (−5.239)
PolyPhen-2	D (0.999)	D (0.993)
MutationTaster	D (0.986)	D (0.999)
Functional domain of protein	no	yes: EF-hand domain 2

A summary of both candidate causal variants includes conservation scores (PhyloP and PhastCons), in silico predictions of pathogenicity (SIFT, PROVEAN, PolyPhen-2, and MutationTaster), and the location of the variant with respect to predicted functional domains of the protein (from UniProt). “Previously reported” refers to the whether the variant can be found in dbSNP, the NHLBI Exome Sequencing Project Exome Variant Server, 1000 Genomes, and Complete Genomics 69. Both variants are conserved and predicted to be damaging by all four prediction programs. Actual numerical scores provided by the in silico prediction programs are shown here in parentheses for the sake of completeness, and readers are referred to the programs’ websites for a detailed explanation of their meaning. Abbreviations are as follows: C, conserved; and D, damaging.

## Web Resources

The URLs for data presented herein are as follows:

1000 Genomes, http://www.1000genomes.org/
Complete Genomics, http://www.completegenomics.com/public-data/69-Genomes/
dbSNP, http://www.ncbi.nlm.nih.gov/projects/SNP/
MERLIN, http://www.sph.umich.edu/csg/abecasis/merlin/index.html
MutationTaster, http://www.mutationtaster.org/
NHLBI GO Exome Sequencing Project, https://esp.gs.washington.edu/drupal/
OMIM, http://www.omim.org
PolyPhen-2, http://genetics.bwh.harvard.edu/pph2/
PROVEAN, http://provean.jcvi.org/index.php
RefSeq, http://www.ncbi.nlm.nih.gov/refseq/
SIFT, http://sift.jcvi.org/
UCSC Genome Browser, http://genome.ucsc.edu/

## Supplemental Data

Document S1. Figures S1 and S2 and Table S1

Document S2. Article plus Supplemental Data
