# Supplementary material for: Mutations in HPCA Cause Autosomal-Recessive Primary Isolated Dystonia
Source: Am J Hum Genet. 2015 Mar 19;96(4):657–65. doi: 10.1016/j.ajhg.2015.02.007 (PMC4385177; doi:10.1016/j.ajhg.2015.02.007)
Supplement: Document S1. Figures S1 and S2 and Table S1 [file mmc1.pdf]

**The American Journal of Human Genetics**

**Supplemental Data**

# Mutations in *HPCA* Cause Autosomal-Recessive Primary Isolated Dystonia

**Gavin Charlesworth, Plamena R. Angelova, Fernando Bartolomé-Robledo, Mina Ryten, Daniah Trabzuni, Maria Stamelou, Andrey Y. Abramov, Kailash P. Bhatia, and Nicholas W. Wood**

Figure S1 – Conservation of Both Candidate Variants

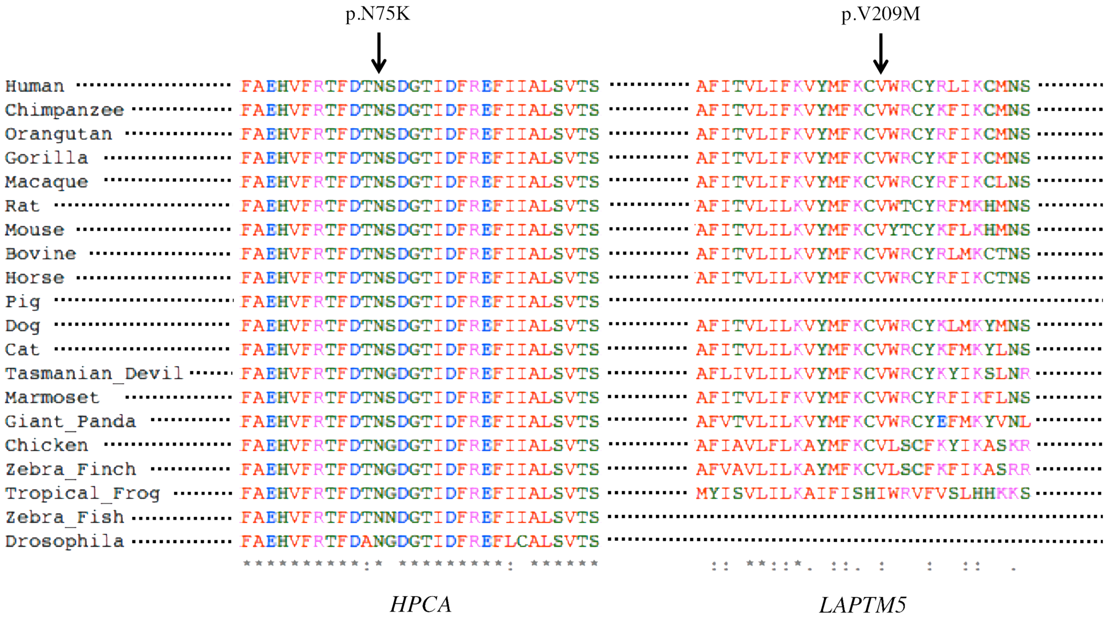


Orthologous protein sequences for the relevant variant-containing regions *HPCA* and *LAPTM5*, obtained via Uniprot and Flybase for all species available and aligned using ClustalOmega. No orthologous sequence was available for worm (*C. Elegans*) in either case. The affected amino acid in the p.Asn75Lys mutation in *HPCA* (left) shows absolute interspecies conservation. Note also the high level of conservation in this region as a whole. The amino acid affected by the p.Val209Met mutation in *LAPTM5* (right) is not fully conserved, with the tropical frog possessing an isoleucine at this position, and no orthologue exists for pig, zebra fish or drosophila. Symbols under each column indicate the degree of conservation (an asterisk = a single identical amino acid; a colon = strongly similar properties; a period = weakly similar properties; a blank = no conservation]. Colours indicate physiochemical properties of amino acids (red = small/hydrophobic; blue = acidic; magenta = basic; green = hydroxyl/sulfhydrl/amine/glycine).

Figure S2 – The Calcium Myristoyl Switch Mechanism

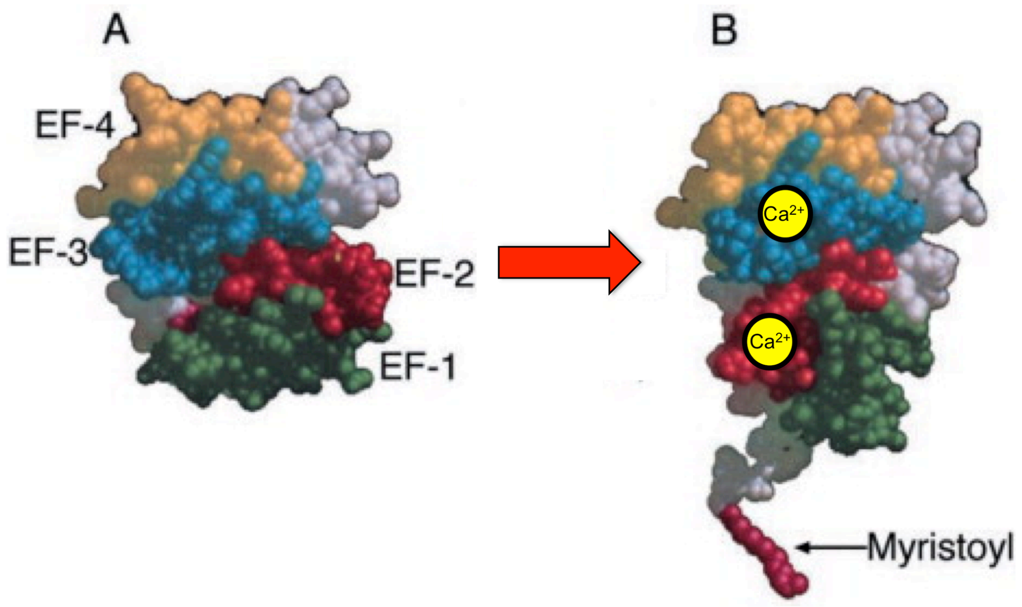


A semi schematic diagram of the calcium-myristoyl switch mechanism using recoverin, a related neuronal calcium sensor protein as an example. A) In the native, non-$Ca^2$+ bound state, recoverin assumes a configuration in which the myristoyl moiety (just visible in magenta) is buried deep within a hydrophobic pocket within the protein. B) Upon binding of $Ca^{2+}$ first to EF-hand domain 3 and then to EF-hand domain 2, the protein undergoes a conformational change that causes the myristoyl moiety to be extruded, freeing it for interaction with target membranes and proteins. (This figure has been modified from Figure 1 as published by Ames et al., 1997)

Table S1 – Publically Annotated Variants in HPCA

Databases of publically available next generation sequencing data from the NHLBI Exome Sequencing Project, ClinSeq, 1000 Genomes and the HapMap project were queried for any SNVs in HPCA expected to affect protein coding (missense, stop gain, stop loss, splicing), regardless of in silico predictions of pathogenicity. In total, this represented a pool of 8,451 individuals (16,902 chromosomes). Only 8 separate missense variants were detected, none of which were common and none of which were homozygous. Only two were predicted to be damaging by both SIFT and PolyPhen.

| **SNP Identifier** | **c.DNA change (NM_002143.2)** | **Protein Change (NP_002134.2)** | **Database of Origin** | **Genotype Count (/8,451)** | **SIFT Prediction** | **PolyPhen Prediction** |
|---|---|---|---|---|---|---|
| Rs11554958 | c.63G>T | p.Glu21Asp | HapMap | G/G = 8,450<br>G/T = 1<br>T/T = 0 | Benign | Benign |
| Rs147332564 | c.178G>C | p.Asp60His | NHLBI ESP | G/G = 8,450<br>G/C = 1<br>C/C = 0 | Damaging | Damaging |
| Rs182483890 | c.196G>A | p.Glu66Lys | 1000 Genomes | G/G = 8,450<br>G/A = 1<br>A/A = 0 | Benign | Damaging |
| Rs201850746 | c.286C>T | p.Arg96Cys | ClinSeq | C/C = 8,450<br>C/T = 1<br>T/T = 0 | Damaging | Benign |
| Rs138767632 | c.373G>A | p.Val125Met | NHLBI ESP | G/G = 8,449<br>G/A = 2<br>A/A = 0 | Damaging | Damaging |
| Rs376349097 | c.403G>T | p.Val135Leu | NHLBI ESP | G/G = 8,450<br>G/T = 1<br>T/T = 0 | Benign | Benign |

| | | | | | | |
|---|---|---|---|---|---|---|
| Rs140440243 | c.412A>C | p.Met138Leu | NHLBI ESP | A/A = 8,450<br>A/C = 1<br>C/C = 0 | Benign | Benign |
| Rs371851892 | c.484G>A | p.Gly162Ser | NHLBI ESP | G/G = 8,450<br>G/A = 1<br>A/A = 0 | Benign | Benign |
